# Clinical implications of germline mutations in breast cancer: *TP53*

**DOI:** 10.1007/s10549-017-4531-y

**Published:** 2017-10-16

**Authors:** Katherine Schon, Marc Tischkowitz

**Affiliations:** 1 East Anglian Medical Genetics Service, Cambridge University Hospitals NHS Trust, Cambridge, UK; 2 Department of Medical Genetics and National Institute for Health Research Cambridge Biomedical Research Centre, University of Cambridge, Cambridge, UK; 3 Department of Medical Genetics, Addenbrooke’s Treatment Centre, Cambridge Biomedical Campus, Box 238, Level 6, Cambridge, CB2 0QQ UK

**Keywords:** *TP53*, Gene panel testing, Li–Fraumeni syndrome

## Abstract

**Purpose:**

This review describes the prevalence of germline *TP53* mutations, the risk of breast cancer and other cancers in mutation carriers and management implications for women with breast cancer and unaffected women.

**Methods:**

Literature review of English language papers available through PubMed.

**Results:**

Women who carry germline mutations in the *TP53* gene have a very high risk of breast cancer of up to 85% by age 60 years. Most of these breast cancers are early onset with a median age at diagnosis of 34 years. Approximately 5–8% of women presenting with breast cancer under 30 years old have a germline *TP53* gene mutation. Breast cancers in women with *TP53* mutations are more likely to be hormone receptor positive and/or Her2 positive. Mastectomy is recommended over lumpectomy in *TP53* mutation carriers who have breast cancer so that adjuvant breast radiotherapy can be avoided. Risk-reducing surgery should be considered due to the high contralateral breast cancer risk. Mutation carriers are at high risk of various childhood and adult-onset cancers with a very lifetime risk of malignancy, the commonest malignancies being breast cancer and soft tissue sarcoma. In unaffected female mutation carriers, MRI breast screening or risk-reducing surgery is recommended. The optimal surveillance for other cancers is currently unclear and should ideally be performed as part of a clinical trial.

**Conclusions:**

Identifying a *TP53* mutation in a gene panel test is a challenging result for the patient and clinician due to the high risk of second primaries and the lack of consensus about surveillance.

## Introduction

Breast cancer gene panel testing has become widely available for women who have been diagnosed with breast cancer and for unaffected women who are concerned about a strong family history of breast cancer. Mutations in the *BRCA1* and *BRCA2* genes remain by far the most common genetic explanation for a strong family history of breast cancer. Germline mutations in *TP53* may cause an even higher risk of breast cancer, but these are much rarer than *BRCA1*/*BRCA2* mutations.

The *TP53* gene is a crucial tumour suppressor gene which has been called ‘the guardian of the genome’. The cellular tumour antigen p53 protein acts as a checkpoint control following DNA damage. It either activates downstream genes to repair the damage or initiates apoptosis. Somatic mutations in *TP53* occur very commonly in the formation of many cancer types and were found in 42% of samples from 12 different cancer types in the Pan-Cancer cohort [1], making it the most frequently mutated gene.

Germline mutations in the *TP53* gene cause a familial cancer predisposition. The syndrome was first observed in 1969 by Li and Fraumeni who described four families of children with soft tissue sarcomas [2]. Mutation carriers have a very high lifetime risk of malignancy and the commonest cancers are soft tissue sarcomas and breast cancer in women. Depending on the pattern of cancers in a family, it may be described as having Li–Fraumeni Syndrome (LFS) (OMIM 151623), Li–Fraumeni-like syndrome (LFL) or it may not meet the diagnostic criteria for these. Various sets of diagnostic criteria and testing criteria have been developed (see table).

**Table:** 

Classic Li–Fraumeni syndrome [3]	All of the following Proband with sarcoma diagnosed before age 45 years A first-degree relative with any cancer before 45 years A first or second-degree relative with any cancer before 45 years or a sarcoma at any age
Li–Fraumeni-like syndrome—Birch [4]	All of the following Proband with any childhood cancer, or a sarcoma, brain tumour or ACC with onset < 45 years A first or second-degree relative with a core LFS cancer (sarcoma, breast cancer, brain tumour, ACC or leukaemia) with onset at any age A first or second-degree relative with any cancer before age 60 years
Li–Fraumeni-like syndrome—Eeles [5]	Two first-degree or second-degree relatives with core LFS malignancies (sarcoma, premenopausal breast cancer, brain tumour ACC, leukaemia, lung [bronchoalveolar] cancer) at any age
Revised Chompret criteria [6]	Proband with a cancer belonging to the Li–Fraumeni spectrum before age 46 AND at least one first- or second-degree relative with a LFS tumour (except breast cancer if proband has breast cancer) before age 56 or with multiple tumours OR Proband with multiple tumours, two of which belong to LFS spectrum and first before age 46 years OR Proband with adrenocortical carcinoma or choroid plexus tumour, regardless of family history

## Prevalence of mutations

### General population

The frequency of germline pathogenic variants in *TP53* in the general population is unknown and has been estimated using penetrance figures. The estimates vary between 1 in 5000 [7] and 1 in 20,000 [8]. Gonzalez and colleagues estimated the frequency of *TP53* germline mutations in the general population using the frequency of specific cancers (breast cancer age ≤ 30 years and adrenocortical carcinoma) in the general population and the frequency of that cancer being due to *TP53* germline mutations. This gave an estimated frequency of 1 in 17,000 to 1 in 23,000 people. A recent study of germline variation in cancer-susceptibility genes in a healthy cohort found 15 *TP53* missense variants and did not find any nonsense or frameshift variant in 681 individuals [9]; one missense variant was likely pathogenic and the others were variants of unknown clinical significance.

### Prevalence of mutations in women with early onset breast cancer

The prevalence of *TP53* mutations among women with early onset breast cancer has been studied in various populations [10, 11]. McCuaig et al. estimated that 5–8% of women with breast cancer diagnosed under age 30 years and no pathogenic variant in *BRCA1* or *BRCA2* will have a pathogenic variant in *TP53,* and a smaller proportion of women with breast cancer diagnosed aged 30–39 years [12]. The likelihood of having a *TP53* mutation is increased if there is a family history of LFS-related cancers, or a personal history of an additional LFS-related cancer.

In a series of patients who had a germline *TP53* mutation ascertained due to having a young onset cancer, it was estimated that 7–20% of the mutations were *de novo* [13]. This is in contrast to hereditary breast and ovarian cancer syndrome due to *BRCA1* or *BRCA2* gene mutations, where *de novo* mutations are exceedingly rare. This observation supports testing very young onset breast cancer patients for *TP53*, even in the absence of family history.

### Prevalence of *TP53* mutations in women who have breast cancer gene panel testing

The prevalence of *TP53* mutations among women who have had panel testing is low at under 1% in four recent studies. Buys et al. found 61 women with *TP53* mutations among 35,409 women with breast cancer who had testing using a panel of 25 cancer genes (0.17%) [14]. Moran et al. detected one *TP53* mutation among 190 breast cancer patients with a strong family history and previous negative *BRCA1*/*BRCA2* testing using a protein truncation test (0.53%) [15]. Kapoor et al. found one *TP53* mutation among 377 women who were offered gene testing by breast surgeons using multigene panels (5–43 genes, average 14.7) (0.27%) [16]. Susswein et al. reported results for over 10,000 consecutive cases referred for evaluation of germline cancer genes. They reported nine pathogenic and one likely pathogenic *TP53* mutation among 3315 women with breast cancer who had not had previous *BRCA1*/*BRCA2* testing (0.30%), and three pathogenic and one likely pathogenic *TP53* mutation among 1894 women with breast cancer who had previous *BRCA1*/*BRCA2* testing (0.21%) [17].

## Founder mutations

There is a high prevalence of the c.1010G > A, p.(Arg337His) mutation in exon 10 (often referred to as R337H) in Southeast and Southern Brazil [18]. Individuals with this mutation have a similar lifetime cancer prevalence to other LFS carriers (about 90%) but a lower penetrance at young ages (< 20% at age 30 years, compared to 50% in other LFS carriers) [19]. It has been suggested that women affected with breast cancer under the age of 45 years in Southeast and Southern Brazil should be offered testing for this mutation, irrespective of family history [20]. The R337H mutation is not common among women diagnosed with breast cancer in Portugal [21]. We are not aware of any other founder mutations in the *TP53* gene.

## Penetrance of breast cancer

The penetrance of breast cancer in women with *TP53* mutations is very high with a cumulative incidence of 85% by age 60 years in the National Cancer Institute Li–Fraumeni Syndrome cohort [22]. The annual hazard for female breast cancer started to increase in the late teens and peaked at approximately 40 years. This was a highly selected cohort of 286 individuals from 107 families. Most met the criteria for LFS (43%) or Li–Fraumeni-like syndrome (38%). 8% had ≥ 3 primaries and 10% had tested positive for a *TP53* mutation without meeting any of the current diagnostic criteria or testing guidelines. Another study found that the median age of onset of breast cancer was 34 years [23]. The lifetime risks in individuals without a strong family history might be lower.

## Range of cancer sites implicated

The overall lifetime risk of cancer in individuals with *TP53* mutations is very high. Wu et al. estimated that the cancer-free survival probabilities for female *TP53* mutation carriers were 65.2, 33.0 and 2.9% at ages 30, 45 and 60 years, respectively. The corresponding cancer-free survival rates for male carriers were 83.4, 62.5 and 22.2% [24]. This gender difference is primarily the result of the high incidence of breast cancer among women with LFS.

There are a wide variety of cancer sites implicated in LFS. LFS was originally described in the families of children with soft tissue sarcomas [2], the second commonest cancer diagnosis (after breast cancer) in the National Cancer Institute Li–Fraumeni Syndrome cohort [22]. The ‘core’ cancers described were sarcomas, breast cancer, adrenocortical cancers and brain tumours. In a cohort of 525 patients tested, 9/9 patients with choroid plexus tumours had an identifiable *TP53* mutation [8]. Other cancers become commoner in older *TP53* mutation carriers, such as lung, colorectal and prostate cancer. Leukaemias and lymphomas can also occur but are not cardinal features.

There is a high risk for multiple primary cancers and a study of 200 individuals from 24 LFS families found that 15% of individuals developed a second cancer, 4% had a third cancer and 2% had a fourth. The cumulative probability of a second cancer occurrence at 30 years after the first cancer was estimated as 75% (± 10%) [25].

Ruijs and colleagues provide estimates for the relative risk of different cancer types in LFS [26]. They found that the highest relative risks were for bone cancers (RR 107, 95% confidence intervals 49–203), connective tissue cancers (RR 61, CI 33–102) and brain tumours (RR 35, CI 19–60). The relative risk for breast cancer was 6.4 (95% confidence intervals 4.3–9.3).

## Determining the pathogenicity of variants

The *TP53* gene is located on chromosome 17p13.1 and encodes the cellular tumour antigen p53. The gene has 11 exons and is 20 kb in genomic length. The coding region encompasses exons 2–11 while exon 1 is non-coding and contains two transcriptional start sites. The majority of pathogenic variants are missense variants (73% in the study by Olivier and colleagues [23]) or small 1–4 bp deletions [23]. There are mutational hot spots at codons 133, 175, 213, 220, 245, 248, 273, 282 and 337 [27]. These codons are also mutational hotspots in sporadic tumours. Many of these are within exons 5–8 which encode the core DNA-binding region of the gene.

95% of mutations can be detected by sequence analysis of all exons [28]. Only approximately 1% of mutations are deletions or duplications involving the coding region, exon 1 or the promoter [28]. A functional assay may be useful in determining the clinical significance of novel pathogenic missense variants but this is only performed in certain research laboratories.

There are several databases with curated information on *TP53* variants, including the p53 Mutation in Human Cancer (http://p53.free.fr/) and the IARC *TP53* Database (http://p53.iarc.fr). A recent paper by Bouaoun [27] used the IARC database to provide an update on *TP53*-inherited variants, including those that should be considered as neutral frequent variants.

### Genotype–phenotype correlations

There have been several studies looking at genotype–phenotype correlations in families with LFS and LFS-like syndrome. Birch and colleagues’ study of 34 families (20 LFS and 14 LFL) showed that individuals with missense mutations in the DNA-binding region had higher overall rates of cancer with significantly higher rates of breast cancer and central nervous system tumours compared to individuals with missense mutations in other parts of the gene or protein-truncating mutations [29]. A study by Olivier et al. analysed the IARC database (including 1068 individuals from 265 families) and found that missense mutations outside the DNA-binding region are more commonly associated with adrenocortical carcinoma compared to missense mutations in the DNA-binding domain. They also noted that individuals with missense mutations in the DNA-binding domain were more likely to have early onset breast cancer compared to those with missense mutations outside the DNA-binding domain (32 years vs. 42 years) and that mutations leading to a *TP53* null phenotype are associated with earlier onset brain tumours [23].

## Management implications

### Management of risk of breast cancer

The option for risk-reducing bilateral mastectomy or breast screening should be considered in women without cancer with a mutation in the *TP53* gene. In the UK, annual MRI breast screening is recommended from age 20 to 49 years and should be considered between 50 and 69 years. Mammography is not recommended [30]. In the USA, National Comprehensive Cancer Network guidelines recommend annual breast MRI 20–29 years and annual MRI and mammography from 30 to 75 years [31]. In Australia, national guidelines recommend that bilateral mastectomy should be offered, otherwise annual breast MRI is recommended from 20 to 50 years [32]. Based on the finding that breast cancer risk increases significantly after the second decade [22], bilateral mastectomy should be considered from age 20 (in line with NCCN guidelines [31]).The annual breast cancer risk peaks at around age 40–45 and then decreases [22]. Bilateral mastectomy is less likely to benefit women over 60 years.

### Management of breast cancer

Mastectomy rather than lumpectomy is recommended to reduce the risks of a second primary breast cancer and to avoid radiotherapy where possible. Bilateral mastectomy should also be considered due to the risk of a contralateral breast cancer. Contralateral breast cancer risk will depend on the patient’s age, but there are no clear figures from the literature.

There are concerns about increased risk of radiation-induced second primary tumours. Heymann et al. [33] describe a series of 8 patients with germline *TP53* mutations who were treated for breast cancer between 1997 and 2007 from 47 documented Li–Fraumeni families; three underwent conservative breast surgery with post-operative radiotherapy, three had mastectomy and radiotherapy and two had mastectomy with no radiotherapy. Among the six who received radiotherapy, there were three ipsilateral breast recurrences, three contralateral breast cancers, two radio-induced cancers (one breast histiocytoma fibrosarcoma and one chest wall angiosarcoma) and three new primaries (including one papillary thyroid carcinoma which developed inside the radiation field after 2 years). One contralateral breast cancer occurred in the two patients who did not have radiotherapy, with a median follow-up of 6 years. Despite the small sample size, this study does suggest that radiotherapy should be avoided or used with extreme caution in *TP53* mutation carriers after very careful consideration of the risks and benefits.

A study of the breast cancer histopathology in *TP53* carriers showed that most invasive ductal carcinomas and ductal carcinomas in situ (DCIS) in LFS are hormone receptor positive and/or HER-2 positive [34]. In this study, there were 32 invasive ductal carcinomas in 30 women with confirmed germline *TP53* mutations. 84% of the tumours stained positive for oestrogen receptors, 72% for progesterone receptors and 63% showed HER-2 amplification and/or overexpression. Melhem-Bertrandt et al. [35] have also reported that women who are *TP53* mutation carriers are more likely to have HER2 amplification and/or overexpression (present in 67% of cases and 25% of controls in this study).

These histopathological findings have management implications as treatment is more likely to include Trastuzumab (Herceptin) and hormone therapy. There are no published data on treatment response to chemotherapeutic agents so standard chemotherapy regiments should be used.

### Management of risks of other cancers

There is no international consensus about the best surveillance for *TP53* mutation carriers. In the UK, breast MRI screening is the only recommendation. In the USA, annual complete physical examination including neurologic and skin examination is recommended, and 2–5 yearly colonoscopy from age 25 [31]. In the Netherlands, annual breast surveillance from age 20 to 25 and an optional annual physical examination is recommended [36]. The Australian guidelines recommend annual clinical review, and to consider 2–5 yearly colonoscopy from 25 years if there is a family history of colorectal cancer, or 2–5 yearly endoscopy if there is a family history of gastric cancer [32].

An intensive and comprehensive surveillance programme has been proposed by Villani et al. for children and adults who carry a *TP53* germline mutation [37]. This uses multiple modalities (physical examination, blood tests, ultrasound, mammography, MRI and colonoscopy). The results of a non-randomised trial showed good long-term compliance with the protocol. It reported a significantly improved five-year overall survival in the surveillance group. However, there was a very high rate of symptomatic tumours in the group who initially declined surveillance. A larger-scale randomised controlled trial is required to evaluate the protocol further.

Neonatal testing for the c.1010G > A, p.(Arg337His) mutation and subsequent surveillance for adrenocortical tumours in mutation carriers has been evaluated in a large, non-randomised clinical trial in Southern Brazil [38]. Adrenocortical tumours diagnosed in the surveillance group were smaller and had an improved clinical outcome. Although the results are not directly transferrable to other populations of *TP53* carriers with different mutations, it does suggest that surveillance for adrenocortical tumours in children should be considered.

The UK SIGNIFY study [39] investigated the role of one-off whole body non-contrast MRI screening in asymptomatic *TP53* mutation carriers. Four malignancies were diagnosed among 44 *TP53* mutation carriers and none in matched controls. Two malignancies were not picked up on scan—one patient developed leukaemia and one became symptomatic from a mediastinal liposarcoma which was thought to be a pericardial cyst on scan. The difference between cancer detection in carriers and controls was not statistically significant, but the trend of the results suggests that whole body MRI is a useful investigation.

### Ongoing clinical trials

There are several ongoing trials using whole body MRI screening (reviewed in McBride 2014 [40] and Ballinger 2015 [41]). The LIFSCREEN trial [42] at the Institut Gustave Roussy (Villejuif, France) is a randomised trial with annual whole body MRI screening for three years in the intervention arm and standard care in the control arm. It is recruiting individuals with *TP53* mutations (age 5–71 years). The Surveillance of Multi-Organ Cancer Prone Syndrome (SMOC) trial [43] in Australia is enrolling individuals with a germline mutation in a cancer risk gene (including *TP53*) and those at 50% risk aged 18–70 years for a period of 3 years. It is a non-randomised trial using annual physical examination, full blood count, whole body MRI, breast MRI, ultrasound and mammography (in females) with additional procedures of gastroscopy and colonoscopy based on family history. The Dana Faber Cancer Institute whole body MRI study involves annual physical examination and whole body MRI and is currently recruiting children only.

Based on the observation that metformin has been associated with reduced cancer risk in several epidemiological studies [44], a pilot study for chemoprevention using metformin has been commenced which assesses the safety and tolerability of the drug over 14 weeks, measuring IGF-1, insulin and IGFBP3 levels in blood at baseline, and weeks 0 and 8 [45].

As the optimal screening for other cancers in *TP53* mutation carriers remains unclear, screening should ideally be undertaken as part of clinical trial.

### Example

A fifty-year old unaffected woman with no family history has a *TP53* pathogenic variant detected on panel testing. The evidence is that this scenario will be very unusual, so we would advise reviewing the literature about the pathogenicity of the specific variant, and we would consider repeating the test (in case of a sample error). We would advise that she is at high risk of developing breast cancer, although she has ‘lived through’ a substantial part of that risk. We would advise annual MRI breast screening or bilateral mastectomy. We would also consider a baseline whole body MRI, based on the UK SIGNIFY study results.

### Support groups

Some women may require additional psychological support. There are several patient support groups including the Li–Fraumeni Syndrome Association <http://www.lfsassociation.org> based in the USA, and the George Pantziarka *TP53* Trust <http://www.tp53.co.uk> based in the UK.

## Conclusions

Finding a germline *TP53* mutation in a woman with breast cancer has significant clinical implications for the patient and her family. Mastectomy is preferred in the treatment of breast cancer so that radiotherapy can be avoided or minimised. Risk-reducing contralateral mastectomy should also be considered, otherwise MRI breast screening should be initiated. The risk of developing second or third primary malignancies is high, and optimal surveillance is currently unclear. Where possible, screening should be undertaken as part of a clinical trial.
